# Functional differences in mesenchymal stromal cells from human dental pulp and periodontal ligament

**DOI:** 10.1111/jcmm.12192

**Published:** 2014-01-03

**Authors:** Anoop Babu Vasandan, Shilpa Rani Shankar, Priya Prasad, Vulugundam Sowmya Jahnavi, Ramesh Ramachandra Bhonde, Susarla Jyothi Prasanna

**Affiliations:** School of Regenerative Medicine (SORM), Manipal University Bangalore, India

**Keywords:** mesenchymal stromal cells, dental pulp stromal cells, pro-inflammatory cytokines, immune properties, periodontal ligament stromal cells, differentiation, stem cells

## Abstract

Clinically reported reparative benefits of mesenchymal stromal cells (MSCs) are majorly attributed to strong immune-modulatory abilities not exactly shared by fibroblasts. However, MSCs remain heterogeneous populations, with unique tissue-specific subsets, and lack of clear-cut assays defining therapeutic stromal subsets adds further ambiguity to the field. In this context, in-depth evaluation of cellular characteristics of MSCs from proximal oro-facial tissues: dental pulp (DPSCs) and periodontal ligament (PDLSCs) from identical donors provides an opportunity to evaluate exclusive niche-specific influences on multipotency and immune-modulation. Exhaustive cell surface profiling of DPSCs and PDLSCs indicated key differences in expression of mesenchymal (CD105) and pluripotent/multipotent stem cell–associated cell surface antigens: SSEA4, CD117, CD123 and CD29. DPSCs and PDLSCs exhibited strong chondrogenic potential, but only DPSCs exhibited adipogenic and osteogenic propensities. PDLSCs expressed immuno-stimulatory/immune-adhesive ligands like HLA-DR and CD50, upon priming with IFNγ, unlike DPSCs, indicating differential response patterns to pro-inflammatory cytokines. Both DPSCs and PDLSCs were hypo-immunogenic and did not elicit robust allogeneic responses despite exposure to IFNγ or TNFα. Interestingly, only DPSCs attenuated mitogen-induced lympho-proliferative responses and priming with either IFNγ or TNFα enhanced immuno-modulation capacity. In contrast, primed or unprimed PDLSCs lacked the ability to suppress polyclonal T cell blast responses. This study indicates that stromal cells from even topographically related tissues do not necessarily share identical MSC properties and emphasizes the need for a thorough functional testing of MSCs from diverse sources with respect to multipotency, immune parameters and response to pro-inflammatory cytokines before translational usage.

## Introduction

Since the advent of bone marrow stromal cells (BMSCs) as off-the-shelf cells for cellular therapy, there had been a hurried need for a more easily accessible stromal stem cell/progenitor cell replacement, which does not involve invasive processing. Mesenchymal stromal cells (MSCs) have now been obtained and identified from most tissues by extrapolating the ‘gold standard’ criteria christened for BMSCs. Despite these claims, the MSC phenotype still remains very confounding, defined minimally by absence of haematopoietic markers, the presence of a set of mesenchymal markers and multi-lineage differentiation potential into connective tissue lineages. These criteria were set by the International Society for Cellular Therapy (ISCT) to ascertain uniformity for defining the MSC phenotype across different culture conditions, isolation procedures and tissue sources 1. However, increasing evidence substantiates the fact that these definitions are not strictly followed for all tissue-derived MSCs with differences in the functional potency within the mesenchymal lineages, even though the basic surface phenotype remains conserved 2–5. *Ex vivo* expanded MSCs are heterogeneous subsets, with respect to their stage of maturity/self-renewal along the differentiation hierarchy. This differentiation hierarchy could be influenced by tissue-specific niches, where MSCs are acclimatized *in vivo*. The dental pulp mesenchyme is surrounded by rich vasculature and is termed ‘ecto-mesenchyme’, owing to its early neural-crest origin, whereas periodontal tissue is in close contact with the dentin and the alveolar bone 6,7. These facets can impact MSC properties. Thus, the present study attempts to evaluate, in-depth, the mesenchymal properties of MSCs derived from geographically nearby dental tissues, dental pulp stromal cells (DPSCs) and periodontal ligament stromal cells (PDLSCs), which *in vivo* occupy different physiological niches. Cosmetic impaction allows for isolation of both DPSCs and PDLSCs from the same individual, negating sample-specific variations, thus providing a unique opportunity to evaluate exclusive niche-specific differences in MSC properties.

Despite change in nomenclature of MSC from multipotent ‘Mesenchymal stem cells’, which have capacity to trans-differentiate to non-mesenchymal lineages, to ‘Mesenchymal stromal cells’, which have limited multipotency restricted to connective tissue lineages, one aspect that is unquestionable is their tremendous healing properties 8. These healing properties have been attributed majorly to immune-modulation, which is induced and influenced by inflammatory stimuli at the site of injury or disease 9. IFNγR1^−/−^ MSCs lack the capacity to correct Graft *versus* host disease (GVHD), further substantiating the role of response of MSCs to inflammatory cytokines in mediating their clinical benefits 10. The immune properties of BMSCs are well studied, but MSCs from dental tissues have not been thoroughly profiled for their immune properties, and their response to pro-inflammatory cytokines, in particular, is not surveyed. As inflammation is an inseparable component in disease and transplantation, one key objective of this study is to assess the immune properties of DPSCs and PDLSCs in presence of key pro-inflammatory cytokines like IFNγ and TNFα before transplantation regimens are planned with these cells.

## Materials and methods

### Isolation of human DPSCs and PDLSCs

Human dental pulp and periodontal ligament tissue were isolated from healthy impacted third molar teeth extracted for cosmetic purposes after obtaining informed consent, as per approved guidelines of the Institutional Ethics committee. The age group of the participants ranged from 17 to 28 years. The tooth was thoroughly washed with dulbecco's phosphate buffered saline (DPBS) (Gibco, Grand Island, NY, USA) containing Antibiotic–Antimycotic (Gibco) and the periodontal ligament tissue was scraped for further processing. The tissue was teased and digested overnight with 0.5 mg/ml Collagenase Blend type H (Sigma-Aldrich, St. Louis, MO, USA) in a 37°C incubator. The digested tissue was washed with DPBS and plated in KO DMEM™ (Gibco) supplemented with 10% FBS (HyClone, Thermo Scientific, Mordialloc, Vic., Australia), 1× GlutaMAX (Gibco) and 1× Antibiotic–Antimycotic. The floating debris was removed after 24 hrs and the adherent cells were allowed to grow till confluence and passaged further in the same medium.

To extract dental pulp from the same tooth, the tooth was cut open with a high-speed dental drill in a sterile environment; the pulp was minced and digested for 3 hrs in 2 mg/ml of Collagenase Blend type H. The digested pulp was washed with DPBS and plated in KO DMEM™ supplemented with 10% FBS, 1× GlutaMAX (Gibco) and 1× Antibiotic–Antimycotic-containing media and allowed to reach confluence before passaging. For all the experiments, cells within passage 6 were used. Comparisons between DPSCs and PDLSCs were carried out at identical passages and from the same tooth tissue to minimize donor-and passage-dependent variations.

### Differentiation of DPSCs and PDLSCs

Confluent MSC cultures were subjected to osteogenic induction in KO DMEM media containing 10 nM Dexamethasone (Sigma-Aldrich), 50 μg/ml of ascorbic acid (Sigma-Aldrich), 10 mM β-glycerophosphate (Sigma-Aldrich), 10% FBS and 1× GlutaMAX. Mineralization was confirmed by Von Kossa staining 11.

Adipogenic ability of MSCs was evaluated by exposing confluent cultures to KO DMEM media with 10% FBS, 1 μM Dexamethasone, 0.5 mM Isobutyl-methyl-Xanthine (IBMX; Sigma-Aldrich), 1 μg/ml Insulin (Sigma-Aldrich) and 100 μM Indomethacin (Sigma-Aldrich). Oil Red O (Sigma-Aldrich) staining was performed to detect oil deposition 11.

Chondrogenic potential was evaluated by culturing confluent MSCs in KO DMEM media with 10% FBS, 1 mM Sodium pyruvate (Gibco), 0.1 μM Dexamethasone, 10 ng/ml of TGFβ1 (Sigma-Aldrich), 50 mg/ml of Ascorbic acid, 1× Insulin-Transferrin-Sodium Selenite pre-mix (Sigma-Aldrich) and 4 mM Proline (Sigma-Aldrich). Chondrogenic induction was assessed by Safranin O staining in monolayer cultures 11.

Mesenchymal stromal cells at passage 4 were used for testing the tri-lineage differentiation potential. Induction media was changed every 4th day till 16 and 18 days before molecular analysis and functional assessment respectively.

### Flow cytometry

Dental pulp stromal cells and PDLSCs between passages 4–6 were trypsinized and re-suspended in DPBS with 0.5% BSA (Sigma-Aldrich) and incubated at 4°C with specific antibodies for 45 min. Cells were subsequently washed in DPBS containing 0.01% azide and fixed with 1% paraformaldehyde and stored at 4°C till further analysis. For basic mesenchymal characterization, mouse anti-human CD73-PE, CD90-PE, CD105-PE, CD34-PE, CD45-FITC, CD19-PE, CD14-PE, HLA-DR-FITC antibody conjugates and the relevant isotype controls were obtained from BD Pharmingen. Detailed characterization for markers expressed on multipotent/pluripotent cell types included staining with mouse anti-human CD117-PE, CD123-PE, CD106-FITC, CD31-PE, SSEA4-FITC, CD13-PE, CD29-FITC and CD9-FITC antibody conjugates and acquisition with a BD LSRII flow cytometer. Percentage positivity for each marker was calculated by gating with respect to the relevant isotype control staining by using the FACS Diva software (BD Biosciences, San Jose, CA, USA).

To check the expression of immune-relevant ligands on DPSCs and PDLSCs upon exposure to pro-inflammatory cytokines, both MSCs were untreated or stimulated with either 150 U/ml of IFNγ (Sigma-Aldrich) or 10 ng/ml of TNFα (Sigma-Aldrich) for 72 hrs, trypsinized and stained with mouse anti-human HLA-ABC-FITC, HLA-DR-FITC, CD80-FITC, CD86-PE, CD95-FITC, CD50-FITC, CD54-PE, CD11a-PE, CD11b-APC and CD166-PE antibody conjugates and the relevant isotype controls (mouse IgG1κ FITC, mouse IgG1κ PE and mouse IgG1κ APC). All antibodies used for flowcytometry analysis were obtained from BD Pharmingen. Cells were acquired on a BD FACS CALIBUR and the overlays were performed with Cell Quest Pro software (BD Biosciences).

### Gene expression analysis in differentiated cultures

Total RNA was extracted from differentiated cultures by using RNeasy PLUS mini kit from Qiagen. 0.5 μg of RNA was reverse-transcribed by using Superscript III First strand synthesis kit (Invitrogen, Carlsbad, CA, USA) as per manufacturer's instructions. Diluted cDNA was amplified by polymerase chain reaction by using specific primer sets in a Veriti 96-well thermo-cycler (Applied Biosystems, Scoresby, Vic., Australia). The cycling conditions included an initializing temperature of 95°C for 5 min followed by denaturation at 94°C for 30 s, annealing at specific annealing temperatures for 30 s, extension at 72°C for 30 s and final extension at 72°C for 10 min. 30-cycle amplification was set to assure the assessment of PCR products at non-saturating PCR conditions for all genes except *SOX9* and *ACAN*. The primers and their specific annealing temperatures are listed in Table 1. mRNA expression of housekeeping gene, *GAPDH*, was used as an internal loading control.

**Table 1:** Primer pairs used to study the tri-lineage differentiation potential of human mesenchymal stromal cells by RT-PCR

Genes	Sequence (5′–3′)	Annealing temperature (°C)
*GAPDH*	S: CGACCACTTTGTCAAGCTCA	50
	A: AGGGGTCTACATGGCAACTG	
*PPARG2*	S: CCATGCTGTTATGGGTGAAA	58
	A: TCAAAGGAGTGGGAGTGGTC	
*LPL*	S: ATGGAGAGCAAAGCCCTGCTC	60
	A: GTTAGGTCCAGCTGGATCGA	
*FABP4*	S: ACCTTAGATGGGGGTGTCCTGGT	64
	A: CGCCTTTCATGACGCATTCCACC	
*RUNX2*	S: GCCCGTGGCCTTCAAGGTGG	60
	A: TCGTCCACTCCGGCCCACAA	
*OCN*	S: CATGAGAGCCCTCACA	52
	A: AGAGCGACACCCTAGAC	
*OPN*	S: TTGCTTTTGCCTCCTAGGCA	61
	A: GTGAAAACTTCGGTTGCTGG	
*SOX9*	S: CGGACACCGAGAACACGCGG	60
	A: GCCTGCGCCCACACCATGAA	
*ACAN*	S: CACTGTTACCGCCACTTCCC	66
	A: ACCAGCGGAAGTCCCCTTCG	
*COL10A1*	S: AGCCAGGGTTGCCAGGACCA	68
	A: TTTTCCCACTCCAGGAGGGC	

### One way mixed lymphocyte reactions

To test the immunogenicity of DPSCs and PDLSCs, 0.1 × 10^5^ (mentioned as +0.1% SC condition) or 0.01 × 10^5^ (mentioned as +0.01% SC condition) mitomycin C-fixed stromal cells between passages 4–6 were co-cultured with 1.0 × 10^5^ allogeneic peripheral blood mononuclear cells (PBMCs) for 6 days. Subsequently, BrdU incorporation assay was performed to check for lympho-proliferative responses elicited against stimulator stromal cells. BrdU uptake was measured by using BrdU cell proliferation assay kit (Calbiochem, San Diego, CA, USA) as per manufacturer instructions. Responses against stimulator mitomycin C-fixed allogeneic PBMCs were used as a positive control for the mixed lymphocyte reaction (MLR) assay.

Relative proliferation index was calculated after normalizing the BrdU uptake of MSCs (DPSCs or PDLSCs) with positive control allogeneic PBMCs (where robust MLR response was noted) from the same donor, which was considered to be 100%.

Stimulator allogeneic PBMCs, as well as stimulator allogeneic MSCs, were fixed with 10 μg/ml of mitomycin C for 2 hrs to arrest proliferation of stimulator cells in the one way MLR. Mitomycin C-fixed cells were washed with DPBS containing 10% FBS before setting up the MLR.

### Mitogen-induced lympho-proliferative responses

For mitogenic stimulation experiments, PBMCs (1.0 × 10^5^ cells/ml) were stimulated with 20 μg/ml of Phytohaemagglutinin (PHA; Biological Industries, Kibbutz Beit-Haemek, Israel) for 72 hrs in the presence and absence of co-cultured mitomycin C-fixed stromal cells at different dose ratios [0.1 × 10^5^ cells/ml (mentioned as +0.1% condition), 0.01 × 10^5^ cells/ml (mentioned as + 0.01% condition)] in 96 well plates and T cell blasts appearing were captured by using a Nikon Eclipse TE2000-U microscope (Melville, NY, USA). Phytohaemagglutinin-induced proliferative responses were quantified by using a BrdU incorporation assay as previously mentioned. Relative proliferation index (RPU) in stromal cell co-cultures was calculated by normalizing the BrdU uptake with PHA-induced PBMC cultures alone, which was considered to be 100%.

For co-cultures involving IFNγ-and TNFα-primed stromal cells and PBMCs, DPSCs or PDLSCs were pretreated with 150 U/ml of IFNγ or 10 ng/ml of TNFα for 72 hrs. The primed MSCs were mitomycin C-fixed, washed and then co-cultured with PHA-stimulated PBMCs at different dose ratios as mentioned above. Mesenchymal stromal cells, both unprimed and primed, were fixed with 10 μg/ml of mitomycin C for 2 hrs, washed and then used for the lympho-proliferation assay.

### Statistical analysis

All values represent the mean values of at least three independent experiments and the corresponding standard error (SEM). Data between different test samples were compared by using Student's *t*-test. A *P* < 0.05 (*) was considered statistically significant and a *P* < 0.001 (**) was considered very significant.

## Results

### Characterization of DPSCs and PDLSCs for mesenchymal and multipotent/pluripotent stem cell–associated antigens

**Fig 1:**
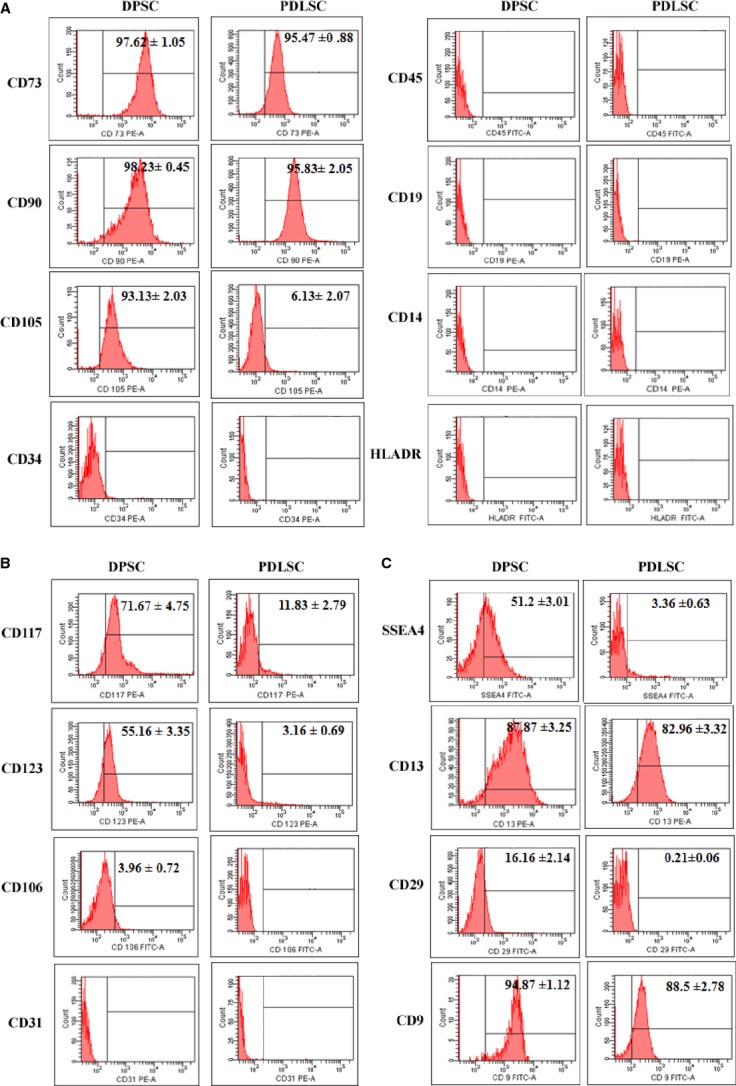
Cell surface phenotype of dental pulp stromal cells (DPSCs) and periodontal ligament stromal cells (PDLSCs). Expression of standard ISCT-defined Mesenchymal markers on DPSC and PDLSC (A). B and C represent a panel of markers known to be expressed on adult and pluripotent stem cell types respectively. Black gates are used to indicate expression above the relevant isotype controls. The values in the histogram represent average% mean positivity ± SE of the specific marker above the isotype control for three independent experiments. % positivity is not mentioned for markers where the expression is below isotype controls.

Stromal isolates from dental pulp and periodontal ligament between passages 3–6 were characterized for markers, as specified by ISCT guidelines 1. Both DPSCs and PDLSCs lacked expression of haematopoietic markers: CD34 (primitive haematopoietic progenitor marker), CD45 (pan-leucocyte marker), CD14 (monocyte/macrophage marker) and CD19 (B cell marker), the absence of which is critical for defining a Mesenchymal stromal cell. HLA-DR, a key marker involved in T cell recognition, was absent in both the MSCs (Fig. 1A). Robust expression of CD73 and CD90 (Thy-1; >95% positivity) was noted in both MSCs. CD105 (Endoglin), a component of the receptor complex of Transforming growth factor-beta, TGFβ, was expressed robustly in DPSCs (>90% positivity), but PDLSCs exhibited a very weak expression pattern (Fig. 1A).

Mesenchymal stromal cells are mostly perivascular with respect to their tissue distribution and bone marrow MSCs and UC-MSCs have been reported to have strong haematopoietic engraftment support abilities 12,13. Thus, multipotent stromal/myeloid progenitors from few sources are reported to express CD117 (SCF R, c-kit), CD123 (IL-3 R) as well as CD106 (VCAM-1) 14,15. Dental pulp stromal cell expressed CD117 and CD123, but only bleak expression of these markers was noted on PDLSCs. Both DPSCs and PDLSCs lack expression of surface antigens present on activated and differentiated endothelial cells like CD106 and CD31 respectively (Fig. 1B). Markers strongly expressed on pluripotent cell types like SSEA4 (stage-specific embryonic antigen-4), CD13 (aminopeptidase N), CD29 (Integrin β1) and CD9 (tetraspanin receptor implicated in early developmental events) were expressed in DPSCs (Fig. 1C). Periodontal ligament stromal cells expressed only CD13 and CD9 and lacked expression of SSEA4 and CD29 (Fig. 1C).

Thus, it appears that stromal cells from dental pulp are probably more similar to well-defined multipotent Mesenchymal/progenitor cells than stromal cells isolated from periodontal ligament with respect to their surface characteristics.

### Tri-lineage differentiation potential of DPSCs and PDLSCs

Dental pulp stromal cells and PDLSCs were tested for their multi-lineage connective tissue differentiation potential by subjecting these cells to adipogenic, osteogenic and chondrogenic induction.

**Fig 2:**
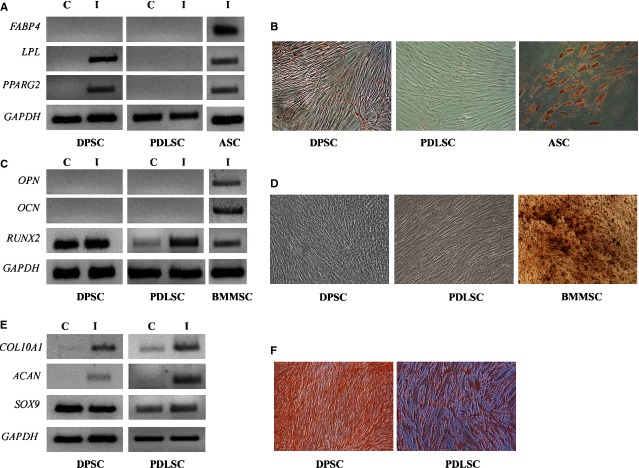
Differentiation potential of dental pulp stromal cells (DPSCs) and periodontal ligament stromal cells (PDLSCs). DPSCs and PDLSCs at passage 4 were subjected to adipogenic (A and B), osteogenic (C and D) and chondrogenic (E and F) induction for 16 days and the cells were characterized for differentiation-specific markers for each lineage by RT-PCR (A, C and E). C refers to control cells unexposed to inductive media and I refers to induced cells. Differentiation to adipogenic, osteogenic and chondrogenic lineages was tested by Oil O Red (B), Von Kossa (D) and Saffranin O (F) staining, respectively, 18 days post-induction. The images are at 200× magnification captured with Nikon Eclipse TE 2000S.

Dental pulp stromal cells as well as PDLSCs did not constitutively express the adipogenic commitment factor, *PPAR*γ*2*, but under adipogenic conditions, induced expression of *PPAR*γ*2* as well as the early adipogenic gene, *Lipoprotein lipase* (*LPL*), was observed in DPSCs (Fig. 2A). Periodontal ligament stromal cells did not express any adipocyte-related genes under adipogenic induction conditions even at the end of 16 days. As compared with control cultures of adipose-derived mesenchymal stem cells (ASC), where robust oil droplet deposition was noted upon adipogenic induction, DPSCs and PDLSCs under identical induction conditions showed sluggish and rare oil deposition and the droplets were smaller and immature (Fig. 2B). This observation correlates with the lack of expression of *Fatty acid binding protein 4* (*FABP4*; a terminal marker associated with mature adipocytes) in differentiated DPSC cultures (Fig. 2A).

Basal expression of *RUNX2*, master transcription factor for osteogenesis, was detected in both DPSCs and PDLSCs. An induction in *RUNX2* levels was noted in PDLSCs upon differentiation, suggesting responsiveness to osteo-inductive factors (Fig. 2C). However, mid and terminal markers associated with osteocytes: *Osteopoientin* (*OPN*) and *Osteocalcin* (*OCN*) were absent in both DPSC and PDLSC differentiation cultures. This was substantiated by lack of mineralization in DPSC and PDLSC cultures in contrast to robust Von Kossa staining of mineral deposits in BMMSC-derived osteogenic cultures (Fig. 2D). A change in morphology of DPSC differentiation cultures was noted at the end of 18 days in induction media (Fig. 2D). Generation of osteoblast and odontoblast precursors engages similar signalling pathways; thus, in the absence of appearance of any terminal osteo-specific markers in DPSCs, it is possible that the change in morphology could be as a result of generation of odontoblast precursors (Fig. S1). Surprisingly, both DPSCs and PDLSCs express *DMP1* (dentin matrix protein 1) and PDLSCs express *DSPP* (dentin sialophosphoprotein), a definitive terminal marker for odontogenesis. In addition, *MSX1* (msh homeobox 1) and *MSX2* (msh homeobox 2), known to be highly induced in pre-odontoblasts and functional odontoblasts, respectively, are induced in both DPSCs and PDLSCs under osteo-inductive condition, suggesting skewing towards dentinogenesis rather than osteogenesis (Fig. S1).

*SOX9*, a transcriptional factor associated with chondrogenesis, was expressed constitutively in both DPSCs and PDLSCs. Upon chondrogenic induction, both DPSCs and PDLSCs expressed the cartilage-specific core proteoglycan, *Aggrecan* (*ACAN*) and α *chain of type X collagen* (*COL10A1*), expressed specifically in chondrocytes during endo-chondrial ossification, suggesting terminal chondrogenesis (Fig. 2E). Further, strong safranin O staining of extracellular matrix was observed in induced cultures of DPSCs and PDLSCs (Fig. 2F).

Thus, DPSCs seem to be more multipotent as compared with PDLSCs, which are unipotent, as they completely lack any adipogenic and osteogenic potential.

### Immune-stimulatory ligand expression on DPSCs and PDLSCs in response to pro-inflammatory cytokines: IFNγ and TNFα

**Fig 3:**
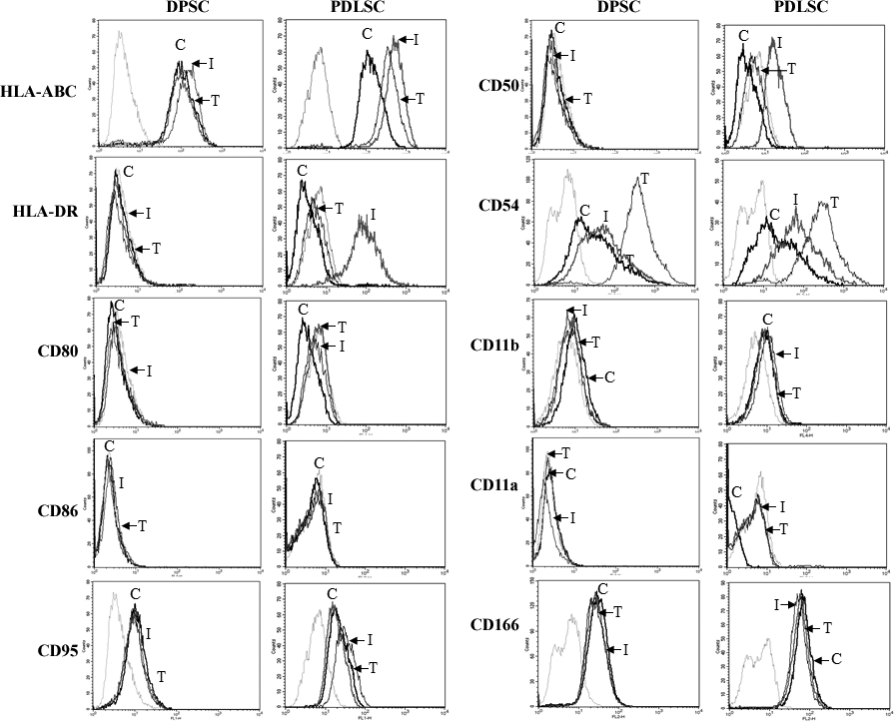
Cell surface expression of immune-stimulatory ligands on dental pulp stromal cells (DPSCs) and periodontal ligament stromal cells (PDLSCs) in response to pro-inflammatory cytokines, IFNγ and TNFα. DPSCs and PDLSCs were treated with 150 U/ml of IFNγ and 10 ng/ml of TNFα for 72 hrs and stained with specific antibodies for flow cytometry analysis. Overlays depict relevant isotype control (Light grey histogram) and specific cell surface expression on untreated control (C), IFNγ-treated (I) and TNFα-treated (T) stromal cells. Data are representative of at least two independent experiments.

Immune recognition involves binding of T cells to allo-antigens, predominated by MHC/HLA antigens and further rejection *via* the indirect pathway, as specified for canonical GVHD. Also productive immune responses are elicited by T cells only after robust signals from co-stimulatory ligands, like CD80/CD86, and immune-adhesion molecules like CD50/CD54 *etc.,* contribute to the formation of a strong immunesynapse 16. Dental pulp stromal cells and PDLSCs express high HLA-ABC like most nucleated cells, and surface levels were induced further with IFNγ or TNFα in PDLSCs and IFNγ in DPSCs. HLA-DR was absent in DPSCs and PDLSCs, but was strongly induced in PDLSCs treated with IFNγ (Fig. 3). No expression of CD80, CD86 and CD11a was noted even after induction with pro-inflammatory cytokines in consensus with published reports in BMSCs 11,17,18. CD166/ALCAM, molecule involved in binding to activated leucocytes, was expressed in both the MSCs, but is not further induced by IFNγ and TNFα. CD54 is constitutively expressed at high levels and levels are further induced by both IFNγ and TNFα in DPSCs as well as PDLSCs. CD50 was not expressed in both the MSCs, but induction was noted upon IFNγ treatment in PDLSCs only (Fig. 3). Bleak expression of CD11b was observed consistently in PDLSCs, but was not induced further by pro-inflammatory cytokines. Fas-FasL signalling is implicated in playing a key role in MSC-mediated immune-modulation 19. CD95 (Fas) was expressed in both MSCs, but was not further induced with inflammatory stimuli (Fig. 3).

In a nutshell, PDLSCs differed from DPSCs in expression of immune-stimulatory ligands and differential response to pro-inflammatory cytokines.

### Immunogenicity of DPSCs and PDLSCs

**Fig 4:**
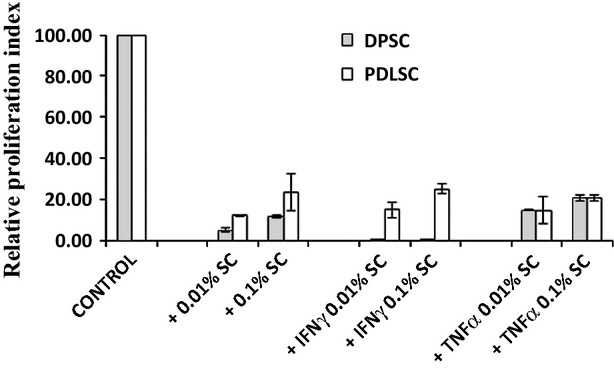
Dental pulp stromal cells and periodontal ligament stromal cells do not evoke a strong immune reaction in one-way mixed lymphocyte reactions. Mitomycin-fixed stromal cells (SC) either primed or unprimed with IFNγ (150 U/ml) or TNFα ng/ml were co-cultured with allogeneic PBMCs at different dose ratios for 6 days and, subsequently, BrdU incorporation was quantified by fluorimetry. Proliferation responses to mitomycinC-treated allogeneic PBMCs were considered 100%. Data are representative of at least two independent experiments.

To check whether DPSCs and PDLSCs evoke an immune reaction from allogeneic immune cells, mitomycin C-treated MSCs were co-cultured with allogeneic PBMCs in a one way MLR. BrdU incorporation was measured to check for any lympho-proliferative responses against allogeneic MSCs at the end of 6 days of co-culture. Mitomycin C-fixed allogeneic PBMCs, used as a positive control, elicited a strong responder PBMC proliferation (considered 100 RPU), whereas no BrdU incorporation was noted with autologous PBMCs. In comparison to allogeneic PBMCs, both DPSCs and PDLSCs elicited only sluggish proliferation (<25% as compared to stimulator allogeneic PBMCs) from allogeneic lymphocytes (Fig. 4). Interestingly, pre-exposure of both the MSCs to inflammatory cytokines, IFNγ and TNFα, also did not enhance immunogenicity despite an increase in immune-stimulatory ligand expression (Fig. 3). Immunogenicity of PDLSCs remained totally unaltered with or without prior exposure to either IFNγ or TNFα. However, ‘IFNγ-primed’ DPSCs did not elicit any proliferative responses from allogeneic PBMCs as compared with unprimed or TNFα-primed DPSCs, which at least elicited some minimal lympho-proliferation response. In totality, both unprimed and primed DPSCs and PDLSCs are hypo-immunogenic under allogeneic conditions, as what is reported for BMSCs 11,17,20,21.

### Immune-modulatory potential of DPSCs and PDLSCs

**Fig 5:**
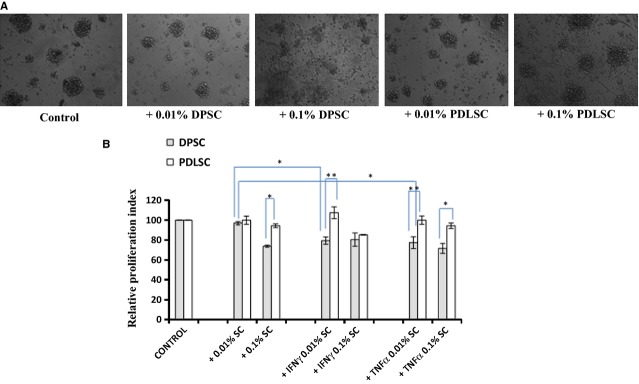
Attenuation of Phytohaemagglutinin (PHA)-induced lympho-proliferative responses by dental pulp stromal cells (DPSCs). Phase contrast images of PHA-stimulated T cell blasts in the presence and absence of co-cultured DPSCs or periodontal ligament stromal cells at 100× magnification after 3 days in culture taken with Nikon Eclipse TE2000S (A). Mean fold proliferation index ± SE of PHA-stimulated T cell blasts co-cultured with different doses of primed or unprimed SCs (+) for at least three independent experiments (B). Proliferative responses of PHA-stimulated PBMCs (Control) are considered 100%.

Peripheral blood mononuclear cells were stimulated with T cell mitogen, PHA, and the polyclonal T cell blast response was assessed in the presence and absence of MSC co-culture. As noted in Figure 5A, DPSCs clearly attenuated the T cell response in a dose-dependent manner. There was no change in the number of blasting cells, but a reduction in the size of the blasts was noted, suggesting suppression of the ongoing response. Surprisingly, PDLSCs did not majorly alter T cell blasts.

T cell blast response was quantitated in the MSC co-cultures by BrdU incorporation and the relative attenuation in proliferation was calculated with respect to the control PHA-stimulated PBMC cultures. Dental pulp stromal cells attenuated PHA-blast response in a dose-dependent manner. Priming of DPSCs with either IFNγ or TNFα further enhanced T cell blast suppression with lower dosage of cells causing attenuation (Fig. 5B). Periodontal ligament stromal cells did not cause any significant attenuation in the T cell blast proliferation, even upon priming with either IFNγ or TNFα. We demonstrate that PDLSCs, unlike DPSCs, do not exhibit any immune-modulatory abilities despite priming with inflammatory cytokines, whereas DPSCs immune-modulate better upon priming with either IFNγ or TNFα.

## Discussion

Mesenchymal stromal cells from healthy adult oral tissues isolated during cosmetic impaction could serve as easily accessible adult MSC source for clinical applications. However, MSCs derived from oro-facial tissues have not been adequately characterized, specifically with respect to their allo-recognition properties most relevant at the transplantation interface. Moreover, a thorough one-to-one comparison of oral MSCs from identical donors for key MSC properties has not been evaluated in detail. An elaborate comparison of DPSCs and PDLSCs for their surface characteristics reveals key differences in the obligate MSC markers as well as a panel of stem cell–related markers (Fig. 1). Of striking notice is the meagre expression of the Mesenchymal marker: CD105 (Fig. 1A), haematopoietic support–related markers: CD117, CD123 (Fig. 1B) and stem cell–associated marker: SSEA4 (Fig. 1C) in PDLSCs as compared with DPSCs, whose profile is similar to bone marrow–derived stromal populations. Low expression of CD105 is reported in heterogeneous periodontal ligament stromal cultures and differences in the proportion of CD105 were noted in deciduous and permanent PDLSCs, with only 11.06% of cells expressing CD105 in adult PDLSCs 22. In addition, CD29, integrin β1, an important component involved in adhering stem cells to their endogenous niches, is absent on PDLSCs (Fig. 1C). As both the MSCs are passage-matched and comparisons have been made from MSCs from same donors, these differences in the surface characteristics could be more intrinsic to the cell type rather than as a result of culture variations, thus obligating the need for a unique phenotypic fingerprint for each stromal cell type.

Intrinsic differences in the tissue regeneration capacity and spontaneous differentiation potential of *in vitro* cultured PDLSCs, DPSCs and BMSCs are reported. When *in vitro* cultured PDLSCs, DPSCs and BMSCs were transplanted subcutaneously in immune-compromised mice, PDLSCs formed cementum-like tissue, DPSCs formed dentin and BMSCs generated typical bone-like structures 23. When a comparative check on the multipotency of DPSCs and PDLSCs was conducted by subjecting these cells to osteo-, adipo-and chondrogenic inductive stimuli, both MSCs robustly responded to chondrogenic stimuli with up-regulation of terminal genes associated with chondrogenesis (Fig. 2E) and chondrocyte-specific matrix deposition (Fig. 2F). However, differences in both osteogenic and adipogenic potential were noted and, markedly, none of the cell types exhibited the robust adipogenic potential and osteogenic potential of ASCs and BMSCs respectively (Fig. 2A–D). Dental pulp stromal cells responded to adipogenic cues and gave rise to cells with immature miniscule droplets, indicating a pre-adipocyte stage, whereas PDLSCs were completely unresponsive to adipogenic stimuli. This observation is in consensus with reports that demonstrated incomplete and sluggish adipogenic potential of DPSCs isolated by different methodologies 24–26. Mineralization and osteogenesis from DPSCs differ markedly from what has been reported with BMSCs, with osteo-induced DPSCs resembling osteo-dentin rather than a typical osteoblast 6. Stem cells from human exfoliated deciduous teeth, considered immature DPSCs, unlike adult DPSCs, have robust osteogenic capacity and also give rise to bone upon *in vivo* transplantation, unlike DPSCs, which give rise to dentin^27^. Periodontal ligament stromal cells have even lesser osteogenic potential than DPSCs and do not form mineralized nodules and calcium depots 23.

In the present study, despite induction of early osteogenic markers under osteogenic conditions, both DPSCs and PDLSCs were detoured towards odontoblast differentiation (Fig. S1). Periodontal ligament stromal cells as well as DPSCs might require additional signalling impetus from pathways like Notch to establish terminal differentiation to osteoblasts 28. Although several reports in the literature indicate osteogenic potential of DPSCs, comparative gene expression studies with osteoblast-like cells generated from DPSCs and normal osteoblasts derived from BMSCs indicated many distinct molecular differences and mineralization patterns resembled osteo-dentin 29. The other reason for unresponsiveness of PDLSCs to osteo-and adipogenic induction could be the absence of CD105; fractionation of PDLSCs based on CD105 sorting has been reported to alter adipogenic potential in adult PDLSCs, emphasizing the need for progenitor fractionation to achieve optimum differentiation 22.

An assessment of detailed immune properties of DPSCs and PDLSCs revealed differences in expression of immune-adhesive ligands and adhesion molecules involved in inflammatory cell recruitment and migration, like CD50 (Fig. 3). Bone marrow stromal cells and other MSCs have been reported to exhibit a hypo-immune profile with total absence of co-stimulatory ligands, CD80 and CD86 expression even upon exposure to pro-inflammatory cytokines, and lack of co-stimulatory support to T cells is one of the main causes for MSC immuneevasiveness 11,17,20,21. In contrast to DPSCs, PDLSCs express immune-adhesive ligands: CD50 and CD11b, co-stimulatory ligand CD80 and HLA-DR, when induced with either IFNγ or TNFα, suggesting that PDLSCs respond to inflammatory cytokine stimulation like fibroblasts and conventional antigen-presenting immune cells. In a MLR, remotely mimicking the *in vivo* allogeneic GVHD response, both IFNγ-/TNFα-primed and unprimed DPSCs as well as PDLSCs do not elicit robust lymphocyte proliferation as reported for BMSCs (Fig. 4). However, DPSCs and PDLSCs differed drastically in their capacity to immune-modulate ongoing T cell blast responses. Priming with either IFNγ or TNFα enhanced immune-modulation capacity of DPSCs (Fig. 5B), as reported for BMSCs 30. This is of particular significance as transplantation of primed DPSCs, rather than naive DPSCs, could be more beneficial to achieve better resolution of inflammation and repair. Surprisingly, PDLSCs, both primed and unprimed, failed to attenuate polyclonal T cell blast responses. In totality, PDLSCs are evidently different from DPSCs as well as other MSCs with respect to response to pro-inflammatory cytokines, expression of immune-stimulatory ligands and their inability to immune-modulate ongoing lympho-proliferative responses. Periodontal ligament stromal cells could be similar to fibroblasts with respect to their inability to immune-modulate, whereas DPSCs are closer to other MSCs with reported immune-modulatory abilities.

There is a huge lacuna in our understanding of genes that pinpoint the exact differences between differentiated fibroblasts and multipotent MSCs, with more than 90% overlap in the surface phenotype of both the cell types. Both immature MSCs and mature fibroblasts coexist in the tissue and isolation procedures based on simple adherence do not distinguish them. In fact, clonal analysis of parent and daughter clones of multipotent MSCs in culture indicates a hierarchical loss of multipotency with emergence of bipotent and unipotent progenitors and ultimate restriction to a fibroblast state 31. Interestingly, recent evidence suggests that even well-characterized, dermal fibroblast lines exhibit some tri-lineage differentiation potential to osteo-, adipo-and chondrogenic lineages. However, fibroblast lines have been shown to lack or exhibit poor immune-suppression/immune-modulation abilities in comparison to MSCs 32. Even the response pattern of MSCs and fibroblasts to inflammatory cytokines with respect to induction of immune-stimulatory ligands differs. Fibroblasts attain antigen presentation ability and pro-inflammatory activity upon exposure to pro-inflammatory cytokines, whereas the immune-suppressive/immune-modulatory activity is further enhanced upon exposure to pro-inflammatory cytokines 32. The surprising lack of immune-modulation ability in PDLSCs, unlike DPSCs and BMSCs 11,17,20, in our study could also be explained because of heterogeneous progenitor subsets differing in immune properties existing within the isolated mesenchymal cells. Of prime importance is the lack of CD105+ population in PDLSCs (Fig. 1A). The expression of CD105 has been correlated with higher multipotency, hepatic trans-differentiation potency in adipose tissue–derived MSCs 33. Similarly amniotic MSCs have been reported to harbour subsets having both immune-suppressive and immune-stimulation abilities depending on the absence and presence of HLA-DR, even though both these populations fail to elicit an allogeneic T cell response 34. Thus, a thorough analysis of immune properties of stromal cells isolated from different tissues could impart an additional handle to demarcate therapeutic multipotent MSCs from fibroblasts, which are co-isolated with MSCs *in vitro*.

In a nutshell, this study indicates that stromal cells from dental pulp and periodontal ligament, although isolated from the same donors, have distinct mesenchymal properties with unique multi-lineage potency, surface characteristics pertaining to existence of unique subsets in the heterogeneity, respond differently to inflammatory stimuli and do not harbour identical immune-modulatory abilities. The identity and function of a ‘universal’ MSC *in vivo* remains an enigma and lack of definitive markers for prospective isolation of MSCs from all sources further adds to the uncertainty 8. Under this scenario, in addition to ISCT-defined functional and phenotypic assessment for MSCs, a thorough evaluation of immune characteristics of MSCs from different niches, akin to that of BMSCs, would help define clinically beneficial MSCs.

## Supporting information

Figure S1 Expression of preodontoblast/odontoblast-associated transcripts in DPSC and PDLSC cultures subjected to osteogenic induction.

Table S1 Primer pairs used to evaluate odontogenic differentiation potential of DPSCs and PDLSCs by RT-PCR.
