# Supplementary figures and images for: Functional differences in mesenchymal stromal cells from human dental pulp and periodontal ligament
Source: J Cell Mol Med. 2014 Jan 3;18(2):344–54. doi: 10.1111/jcmm.12192 (PMC3930420; doi:10.1111/jcmm.12192)

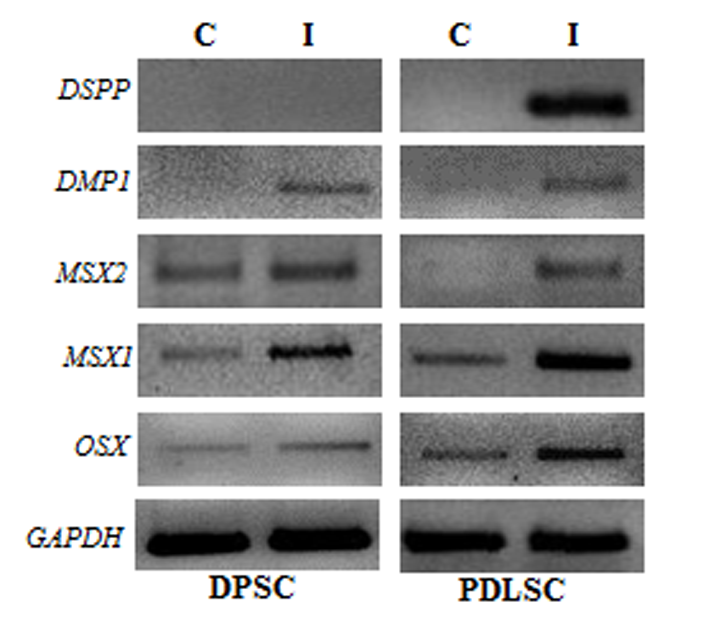

Supplement: Figure S1 — Expression of preodontoblast/odontoblast-associated transcripts in DPSC and PDLSC cultures subjected to osteogenic induction. [file jcmm0018-0344-sd1.tif]
