# Supplementary material for: Functional differences in mesenchymal stromal cells from human dental pulp and periodontal ligament
Source: J Cell Mol Med. 2014 Jan 3;18(2):344–54. doi: 10.1111/jcmm.12192 (PMC3930420; doi:10.1111/jcmm.12192)
Supplement: Table S1 — Primer pairs used to evaluate odontogenic differentiation potential of DPSCs and PDLSCs by RT-PCR. [file jcmm0018-0344-sd2.docx]

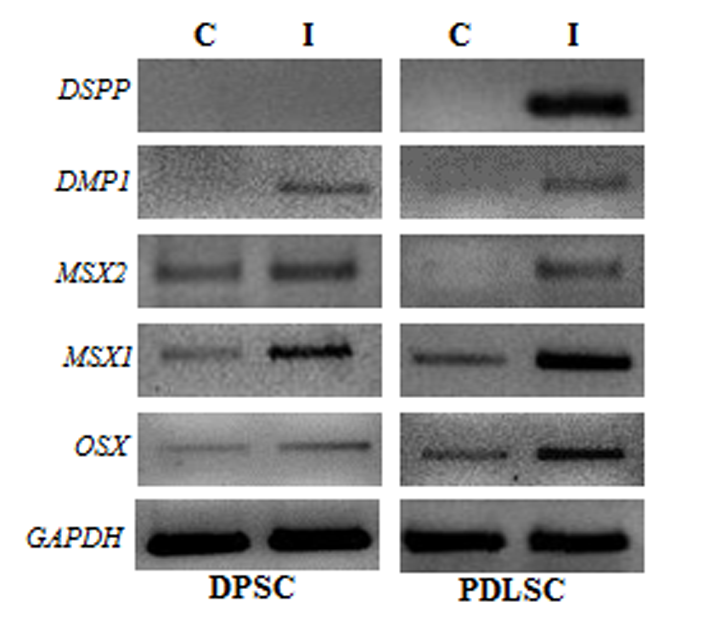


Supplementary Figure 1: **Expression of preodontoblast/odontoblast associated transcripts in DPSC and PDLSC cultures subjected to osteogenic induction. C** and **I** refer to transcript levels in control cells and cells exposed to osteo-inductive media for 16 days respectively. The gel pictures are representative of at least two independent experiments.

**Supplementary Table 1:** Primer pairs used to evaluate odontogenic differentiation potential of DPSCs and PDLSCs by RT-PCR

| **Genes** | **Sequence (5’ -3’)** | **Annealing Temperature**  **(˚C )** |
| --- | --- | --- |
| *OSX* | **S :** GGCACAAAGAAGCCGTACTC  **A :** CACTGGGCAGACAGTCAGA | 59 |
| *MSX1* | **S :** CCTTCCCTTTAACCCTCACAC  **A:** CCGATTTCTCTGCGCTTTTC | 54 |
| *MSX2* | **S:** AGCGGCGTGGATGCAGGAACG  **A:** GCGCGGCTTCCGATTGGTCTG | 60 |
| *DMP1* | **S:** TTGACAATGAGGACCGGGTG  **A:** TCCTGATGCTCTCTGGGTCA | 55 |
| *DSPP* | **S:** CACAGCAAATGGCATCCAGG  **A:** TTGGACAACAGCGACATCCT | 55 |
